# gTranslate: rapid and accurate translation table prediction for prokaryotic genomes

**DOI:** 10.7717/peerj.21825

**Published:** 2026-10-22

**Authors:** Pierre-Alain Chaumeil, Philip Hugenholtz, Donovan H. Parks

**Affiliations:** 1 Center for Microbial Communities, Department of Chemistry and Bioscience, Aalborg University, Aalborg, Denmark; 2 Australian Centre for Ecogenomics, School of Chemistry and Molecular Biosciences, The University of Queensland, Brisbane, Queensland, Australia

**Keywords:** Translation table, Machine learning, Metagenomics, Bioinformatics, Stopreassignment, *Patescibacteriota*, Genetic code

## Abstract

**Background:**

Bioinformatic tools often require the prediction of protein-coding genes to make inferences about prokaryotic genomes. Typically, the genetic code used for translating genes to proteins must be specified by the user based on the taxonomic classification of a genome assembly or, for some widely used tools, established using a heuristic rule based on gene coding densities. Manual specification is at best inconvenient, but more challenging is that many bioinformatic tools are applied before taxonomic classifications have been established making specifying the translation table impractical.

**Methods:**

Here we provide a computationally efficient tool, gTranslate, that uses an ensemble of five machine learning methods to accurately predict translation tables for prokaryotic genomes. The feature vector used by gTranslate takes advantage of differences in gene coding densities when predicting genes under different translation tables along with features that consider the number and ratio of UGA stop codon reassignments to tryptophan or glycine. gTranslate is freely available at https://github.com/cmc-aau/gTranslate and archived on Zenodo.

**Results:**

We demonstrate that gTranslate correctly predicts the translation table of prokaryotic genomes >99.99% of the time (*i.e.*, <1 error per 10,000 genomes) and outperforms a more computationally expensive prediction method and a coding density heuristic used by popular bioinformatic tools. Using gTranslate, we identify a basal lineage of *Ca.* Stammera capleta that uses the standard bacterial genetic code instead of the UGA stop codon to tryptophan reassignment common to other members of this species. We also identify the first instances of UGA-to-tryptophan reassignment in the *Patescibacteriota* making this the first bacterial phylum with members capable of using translation tables 4, 11, and 25.

## Introduction

The genetic code defines the correspondence between nucleotide triplets (codons) in messenger RNA and the amino acids or stop signals they specify during protein synthesis (Koonin & Novozhilov, 2009). Although this code is nearly universal across all domains of life, deviations from the standard code have been documented in both nuclear and organellar genomes spanning eukaryotic, bacterial, and archaeal lineages (Knight, Freeland & Landweber, 2001; Shulgina & Eddy, 2021). These deviations most commonly involve the reassignment of stop codons to amino acids, though sense codon reassignments have also been identified in certain eukaryotic lineages (Mühlhausen & Kollmar, 2014) and have been computationally predicted to occur in some prokaryotes (Shulgina & Eddy, 2021). Despite the diversity of known reassignments, the high conservation of the genetic code across billions of years of evolution underscores the significant selective constraints on its modification and the rarity of successful reassignment events.

While more than 99% of known prokaryotic species use the standard bacterial genetic code *i.e.,* translation table 11, two variant codes have been identified in bacteria involving reassignment of the UGA stop codon. Translation table 4 encodes UGA as tryptophan and is used by all species of the *Bacillota* order *Mycoplasmatales*, a group of endosymbionts primarily associated with vertebrates, arthropods, and plants (Razin, Yogev & Naot, 1998). This reassignment also occurs in several *Pseudomonadota* endosymbionts with highly reduced genomes, including *Candidatus* Hodgkinia cicadicola in cicadas (McCutcheon, McDonald & Moran, 2009), *Ca.* Zinderia insecticola in spittlebugs (McCutcheon & Moran, 2010), *Ca.* Nasuia deltocephalinicola in leafhoppers (Bennett & Moran, 2013), *Ca.* Stammera capleta in tortoise leaf beetles (Salem et al., 2017), and an *Fastidiosibacteraceae* species in dinoflagellates (Nakayama et al., 2025). More recently, this reassignment has been identified in two *Verrucomicrobiota* ciliate endosymbionts *Ca.* Organicella extenuate (Williams et al., 2021) and *Ca*. Pinguicoccus supinus (Serra et al., 2020), and in two genera in the *Actinomycetota* family *Eggerthellaceae* (Parks et al., 2025). Translation table 25 encodes UGA as glycine and was first identified in members of the candidate phylum *Absconditabacteria* (Campbell et al., 2013), and subsequently found in *Gracilibacteria* (Rinke et al., 2013). These bacterial reassignments share several genomic hallmarks, including reduced genome size, compositional bias, and loss of the peptide chain release factor RF2 (McCutcheon, McDonald & Moran, 2009). In addition, many lineages with a stop codon reassignment contain tRNA^Trp^ or tRNA^Gly^ with the anticodon UCA to directly facilitate translation of UGA to tryptophan or glycine, respectively (McCutcheon, McDonald & Moran, 2009; Nakayama et al., 2025). The first genome-wide stop codon reassignment in archaea was recently reported; a UAG-to-pyrrolysine reassignment predicted in multiple archaeal lineages and validated by proteomic evidence (Kivenson et al., 2025).

The genetic code used by an organism can be established from its taxonomic classification, but this presents a practical challenge for bioinformatic workflows that require protein-coding gene predictions before taxonomic classifications have been established. A primary example is GTDB-Tk, which classifies genomes relative to the Genome Taxonomy Database (GTDB) by placing them into a reference tree based on the concatenation of phylogenetically informative protein sequences (Chaumeil et al., 2022). More broadly, prediction of protein-coding genes and their correct translation is an essential step in many common bioinformatic applications, including estimating genome quality (Chklovski et al., 2023; Manni et al., 2021), annotating the function of proteins (Seemann, 2014; Tanizawa, Fujisawa & Nakamura, 2018), and taxonomic profiling of metagenomic samples (Dmitrijeva et al., 2025; Woodcroft et al., 2025). Tools have been developed to detect genetic code reassignments in viral genomes (Pfennig, Lomsadze & Borodovsky, 2023; Cook et al., 2024; Camargo et al., 2024) or from eukaryotic sequence data such as GenDecoder that targets metazoan mitochondria (Abascal, Zardoya & Posada, 2006), Bagheera that predicts CUG codon translation in yeast species (Mühlhausen & Kollmar, 2014), and CoreTracker that detects codon reassignments in mitochondrial genomes using a phylogenetic framework (Noutahi et al., 2017). FACIL and its successor Codetta provide a more general approach by aligning nucleotide sequences against profile hidden Markov models of conserved protein domains to infer the most likely amino acid encoded by each codon (Dutilh et al., 2011; Shulgina & Eddy, 2021). However, while these tools provide evidence of potential codon reassignments, they do not make explicit predictions regarding which translation table a genome uses and are computationally expensive as they require homology searches against large protein databases.

Bioinformatic tools often require users to specify a translation table, which is at best inconvenient and often impractical to determine, resulting in prokaryotic genomes being erroneously processed with the standard bacterial genetic code. This produces artificially truncated and potentially misannotated proteins in lineages with a stop codon reassignment. More recent tools, such as CheckM (Parks et al., 2015), address this issue by using a coding density-based heuristic to predict the translation table of prokaryotic genomes. The CheckM heuristic considers the coding density of genomes using the standard prokaryotic genetic code (CD_11_) and UGA recoded for tryptophan (CD_4_), relying on the observation that genomes recoding UGA to an amino acid will have atypically low coding density when genes are predicted using translation table 11. Specifically, this heuristic uses the decision boundary CD_4_–CD_11_ > 5% and CD_4_ > 70% to establish a genome as using table 4. This approach has been adopted by several widely used tools, including GTDB-Tk (Chaumeil et al., 2022), CheckM2 (Chklovski et al., 2023), and SingleM (Woodcroft et al., 2025). Similar coding density-based strategies are used by BUSCO (Manni et al., 2021) and MiGA (Rodriguez-R et al., 2018).

While coding density-based heuristics generally perform well, even a low misclassification rate becomes problematic for large-scale studies or genomic resources encompassing hundreds of thousands or millions of genomes. Here we introduce gTranslate, a computationally efficient tool that provides improved performance for predicting translation tables for bacteria. We introduce several genomic features that are predictive of bacterial genetic codes and use these to train an ensemble of five machine learning classifiers. Unlike the coding density-based heuristic, which can only distinguish between translation table 11 and a UGA recoding, gTranslate can differentiate between translation table 11, 4, and 25. During evaluation of gTranslate we identified the first members of the genus *Ca.* Stammera to use genetic code 11 instead of 4 and the first members of the phylum *Patescibacteriota* to use genetic code 4. These predictions are supported by additional genomic evidence.

## Materials & Methods

Portions of this text were previously published as part of a preprint (doi: https://doi.org/10.64898/2026.05.24.727570).

### Training and validation data

gTranslate was trained and validated using genomes in the Genome Taxonomy Database (GTDB; Parks et al., 2025)with both GTDB and NCBI taxonomic classifications used to establish the expected ground truth for each genome (see *Establishing the Expected Ground Truth* below). The 596,861 genomes in GTDB R09-RS220 were dereplicated to 356,020 genomes by randomly selecting at most 100 genomes per species to mitigate biasing results to species represented by large numbers of genomes. The ground truth was table 11 for 354,299 (99.52%) genomes, table 4 for 1,521 (0.43%) genomes, and table 25 for 200 (0.06%) genomes (Table S1: https://doi.org/10.5281/zenodo.20761714). These genomes had an average completeness of 92.0% (min. = 18.0%) and contamination of 1.3% (max. = 49.1%) as estimated with CheckM2 1.0.2 (Chklovski et al., 2023).

### Independent test data

The 116,508 genomes in GTDB R10-RS226 not contained in GTDB R09-RS220 were used as an independent test set to evaluate the expected performance of gTranslate (Table S2: https://doi.org/10.5281/zenodo.20761714), with expected ground truth established as for the training and validation data. The ground truth for these genomes was table 11 for 115,940 (99.51%) genomes, table 4 for 447 (0.38%) genomes, and table 25 for 121 (0.10%) genomes. These genomes had an average completeness of 92.7% (min. = 38.0%) and contamination of 1.3% (max. = 22.9%).

gTranslate was also evaluated on 134,075 genomes from GlobDB release 226 (Speth et al., 2024) after removing genomes that: (i) appear in GTDB (143,607 filtered genomes), (ii) have an unresolved classification at the rank of order or higher which precludes establishing an accurate ground truth (754 filtered genomes), or (iii) are <50% complete, >10% contaminated, or <50 quality (completeness-5×contamination) according to CheckM2 estimates (27,822 filtered genomes) (Table S3: https://doi.org/10.5281/zenodo.20761714). The remaining GlobDB genomes come from studies producing large numbers of genome assemblies that have not been submitted to an International Nucleotide Sequence Database Collaboration (INSDC) repository and thus do not appear in any GTDB release. The expected ground truth was established using the taxonomic classifications provided for these genomes by GlobDB, which are determined using GTDB-Tk 2.5.2 (Chaumeil et al., 2022) classifications extended by an automated method for assigning genomes to new taxa. The ground truth for these genomes was table 11 for 133,566 (99.62%) genomes, table 4 for 89 (0.07%) genomes, and table 25 for 420 (0.31%) genomes. These genomes had an average completeness of 85.4% (min. = 50.1%) and contamination of 1.78% (max. = 9.98%).

### Establishing the expected ground truth

The ground truth (*i.e.,* presumed correct) translation table for each genome assembly was established by considering its classification in the GTDB and NCBI taxonomic frameworks (Parks et al., 2026; Schoch et al., 2020). Based on prior information, genomes in the GTDB class JAEDAM01, which contains the two GTDB orders *Absconditabacterales* and BD1-5, were considered to use translation table 25. Note that the GTDB class JAEDAM01 as opposed to class *Gracilibacteria* (Rinke et al., 2013) contains organisms using translation table 25 due to an error in the specification of the type strain, which defines the position of *Gracilibacteria* (*personal communication* with Chris Rinke). Genomes belonging to the GTDB order *Mycoplasmatales* (Razin, Yogev & Naot, 1998), the GTDB species *Ca.* Zinderia insecticola (McCutcheon & Moran, 2010), and the *Eggerthellaceae* genera *Gorillivita* or *Equivita* (Parks et al., 2026) were considered to use translation table 4. Genomes assigned to the NCBI species *Ca.* Hodgkinia cicadicola (McCutcheon, McDonald & Moran, 2009), *Ca.* Nasuia deltocephalinicola (Bennett & Moran, 2013), *Ca.* Stammera capleta (Salem et al., 2017), *Ca.* Organicella extenuate (Williams et al., 2021), and *Ca*. Pinguicoccus supinus (Serra et al., 2020) were also considered to use translation table 4. These species have highly reduced genomes and are not included in GTDB as they lack sufficient phylogenetically informative genes to be robustly placed in the reference trees used by this taxonomic resource. *Fastidiosibacteraceae* strain XS4 (Nakayama et al., 2025), which uses translation table 4, was not included in the analysis as no genome assemblies for the species are currently available in the NCBI Assembly database (Kitts et al., 2016). All other species were considered to use the standard bacterial code (translation table 11).

### Calculation of the feature vector

Genomes were transformed into a vector consisting of eight features that were used by all evaluated machine learning (ML) classifiers:

• *Coding density 11* (CD11): gene coding density when predicting genes with Prodigal (Hyatt et al., 2010)using translation table 11.
• *Coding density 4/25* (CD4): gene coding density when predicting genes with Prodigal using translation table 4 (or equivalently 25), which reassigned the UGA stop coding to either tryptophan (or glycine).
• *Coding density difference*: the difference in coding density between CD4 and CD11 (CD4 –CD11).
• *GC percentage:* percentage of guanine (G) and cytosine (C) nucleotides in a genome.
• *Tryptophan ratio*: log-transformed ratio of UGA to UGG codon counts when predicting genes with Prodigal under translation table 4. The log ratio is clamped between −6 and 5 to remove extreme outliers.
• *Tryptophan magnitude*: log-transformed count of all UGA and UGG tryptophan codons when predicting genes with Prodigal under translation table 4.
• *Glycine ratio:* log-transformed ratio of UGA codon counts to the total glycine codon counts (*i.e.,* codons GGn) when predicting genes with Prodigal under translation table 4. The log ratio is clamped between −10 and 0 to remove extreme outliers.
• *UGG density*: ratio of UGG tryptophan codons to glycine codons (GGn) when predicting genes with Prodigal under translation table 4.

Prodigal was run with default parameters with the exception that (i) proteins were not called across ambiguous bases by using the restricted gene prediction (-m), and (ii) setting the prediction procedure (-p) to ‘meta’ if a genome assembly consisted of <100 kb. While ML classifiers can learn non-linear decision boundaries that utilize all eight features, pairwise plots are sufficient to illustrate that these features can discriminate between translation tables (Fig. S1). Features were normalized between 0 and 1 using min–max scaling for the *k*-nearest neighbours and multi-layer perceptron classifiers.

### Evaluated classifiers and hyperparameter value selection

The translation table for each genome was predicted using seven ML classifiers, the CheckM coding density heuristic (Parks et al., 2015) and Codetta (Shulgina & Eddy, 2021). Specifically, we evaluated AdaBoost, *k*-nearest neighbours (*k*-NN), decision tree, random forest, and multi-layer perceptron (MLP) classifiers as implemented in the Scikit-learn 1.6.1 Python package (Pedregosa et al., 2011), and the LightGBM 4.6.0 (Ke et al., 2017) and XGBoost 2.1.4 (Chen & Guestrin, 2016) gradient boosting classifiers. A grid search was performed over the most salient hyperparameters for each of the classifiers (Table S4: https://doi.org/10.5281/zenodo.20761714) with 5-fold cross-validation across 80% of randomly selected GTDB R09-RS220 genomes used to determine the hyperparameter values resulting in the highest mean accuracy across the folds. Performance of the classifiers using the selected hyperparameter values was then validated using 5-fold cross-validation across the full set of GTDB R09-RS220 genomes to evaluate initial classifier performance and to ensure classifiers did not overfit to the 80% of data available during hyperparameter value selection.

Codetta provides evidence for alternative genetic codes and does not directly provide a prediction for a specific translation table. Specifically, alignments of profile hidden Markov models of conserved proteins to codons are used to identify the most likely translation for each codon. We evaluated the accuracy of Codetta to correctly predict translation table 11, 4, or 25 by examining the evidence provided by Codetta for a UGA stop codon reassignment. We followed the recommendation of Shulgina & Eddy (2021) and evaluated Codetta assuming either eight or 34 columns as sufficient support to treat the UGA stop codon as being reassigned to tryptophan or glycine and thus a prediction of translation table 4 or 25, respectively.

The accuracy of the translation table information provided by NCBI, which is strictly based on taxonomic information (taxon ids), was also assessed. Translation table information was obtained from the GenBank Flat File (GBFF) for each genome when available or the translation table associated with an NCBI species classification. NCBI translation table information could only be established for 207,494 (58.3%) of the 356,020 genomes in GTDB R09-RS220 as many genomes lack an NCBI species assignment (Table S1: https://doi.org/10.5281/zenodo.20761714).

### Ensemble of classifiers

All possible combinations of three and five ML classifiers were formed into an ensemble with majority voting used to establish the final prediction. Ties, while rare, are possible given that there are three possible translation tables. Two types of ties can occur when considering an ensemble of five classifiers. The first involves a tie resulting from two table 11 predictions, two table 4 (25) predictions, and a single vote for table 25 (4). In this case, majority vote predicts a reassignment of the UGA stop codon and the tie is resolved by predicting either table 4 or 25. For example, if the five votes are 11, 11, 4, 4, and 25 the ensemble classifier will predict table 4. The second type of tie can occur when using an ensemble of three or five classifiers and occurs when there are an equal number of votes for table 4 and 25 (*e.g.*, 11, 4, 4, 25, 25). In this case, there is no unambiguous way to resolve the tie between table 4 and 25. Consequently, gTranslate will produce a warning and predict table 4 as this table occurs more frequently in large genome datasets. In practice, this situation is exceedingly rare. It occurred only once across the validation tests with R09-RS220 and did not occur in the final R10-RS226 or GlobDB testing when using the five-classifier ensemble ultimately selected for use in gTranslate.

### Leave-one-taxon-out validation

The performance of the seven ML classifiers was evaluated using a leave-one-taxon-out procedure to validate performance on genomes representing different levels of taxonomic novelty. Specifically, all genomes in a single GTDB taxon were removed from the GTDB R09-RS220 set, classifiers were then trained on the remaining genomes, and prediction results evaluated on the genomes from the removed taxon. This procedure was performed for the ranks of species, genus, and family as removing taxa at broader ranks can result in insufficient training data for translation table 4 and 25.

### Tree inference

Phylogenetic trees were inferred for lineages with consistently different translation table predictions to the ground truth. A *Ca.* Stammera capleta tree was inferred from a set of 181 core genes identified using Panaroo 1.5.2 (Tonkin-Hill et al., 2020) run in strict mode using a sequence identity threshold of 60%, protein family identity threshold of 50%, length difference threshold of 5%, and core gene threshold of 70%. Genes were aligned using MAFFT 7.505 (Katoh & Standley, 2013) run using the L-INS-i alignment method and–maxiterate set to 1,000. A maximum-likelihood tree was inferred with IQ-Tree 2.4.0 (Minh et al., 2020) with the evolutionary model GTR+F+R5 selected using ModelFinder (Kalyaanamoorthy et al., 2017). Support values were determined using 1,000 replicates for both the SH approximate likelihood ratio test (SH-aLRT; Anisimova et al., 2011) and ultrafast bootstraps (UFBoot; Hoang et al., 2018). As in previous studies of this species (García-Lozano et al., 2024), the tree was rooted using *Buchnera aphidicola*.

Phylogenetic trees for *Minisyncoccia* genomes in support of the JAKLIH01 family and GCA-2747955 genus having a UGA stop codon reassignment were inferred using the 120 GTDB bacterial marker genes (bac120). These trees were inferred using GlobDB release 226 genomes with families consisting of >10 genomes dereplicated to the 10 highest-quality genomes based on CheckM2 estimates. Bac120 marker genes were identified, aligned and trimmed using the *de novo* inference workflow of GTDB-Tk 2.5.2 with default parameters. Maximum-likelihood trees were inferred with IQ-Tree 2.4.0 with the evolutionary model LG+F+I+R10 selected using ModelFinder for both trees. Support values were determined with 1,000 replicates for SH-aLRT and UFBoot. Trees were rooted to conform to the GTDB R10-RS226 reference tree topology.

### Calculation of genome properties

Genomes were annotated with Prokka 1.14.6 (Seemann, 2014) using its bacterial reference databases. Aragorn 1.2.38 (Laslett, 2004) was used internally by Prokka to identify tRNA genes. Prokka annotations were inspected to identify tRNA^Trp^(UCA), tRNA^Gly^(UCA) and peptide chain release factor 2 (RF2, *prfB*) genes. %GC content and genome size were determined using CheckM2 1.0.2.

## Results

### Selection of classifier hyperparameter values

A grid search was conducted over the most salient hyperparameters for seven ML classifiers under consideration for inclusion in gTranslate, with performance assessed using 5-fold cross-validation over 284,816 (80%) of the 356,020 GTDB R09-RS220 genomes (Table S4: https://doi.org/10.5281/zenodo.20761714). Classifier performance varied substantially across parameters highlighting the importance of conducting hyperparameter value selection (Fig. 1). However, all classifiers with the exception of MLP had similar performance over a range of hyperparameter values indicating robustness to value selection. The combination of hyperparameter values resulting in the highest accuracy (fewest misclassifications) cumulatively across the five folds was selected for each classifier.

### Classifier performance using cross validation

We evaluated the performance of the seven ML classifiers and 56 ensembles of these classifiers using five-fold cross validation across the 356,020 genomes in GTDB R09-RS220. This includes the 20% of genomes withheld during hyperparameter selection to ensure that the classifiers are generalizing to new genomes. Performance varied across the classifiers with *k*-NN achieving the best results with only 15 misclassifications and the decision tree performing the worst with 30 misclassifications (Fig. 2A; Fig. S2). The best ensemble classifiers were able to reduce the number of misclassifications to 14, with many different combinations of classifiers outperforming all individual classifiers except *k*-NN (Table S5: https://doi.org/10.5281/zenodo.20761714). All classifiers and classifier ensembles had better performance than the CheckM coding density heuristic (101 misclassifications) or Codetta requiring either eight (444 misclassifications) or 34 (78 misclassification) columns supporting a UGA stop codon reassignment (see *Materials & Methods*; Fig. 2A; Table S1: https://doi.org/10.5281/zenodo.20761714). NCBI translation table data was available for 207,495 (58.3%) of the genomes, and 62 of these were found to disagree with the expected ground truth (Table S1: https://doi.org/10.5281/zenodo.20761714). This includes: (i) 28 genomes classified by NCBI as *Candidatus* Gracilibacteria with assembly data indicating table 25, but GTDB assignments to taxa that use table 11, (ii) 16 genomes classified by NCBI only as Bacteria were assigned to table 11, whereas GTDB assigns these taxa to table 4 (*Mycoplasmatales*) or table 25 (JAEDAM01), and (iii) six genomes classified by NCBI to the family *Mycoplasmoidaceae* were assigned to translation table 11, which should be table 4 based on the taxon. Notably, both the CheckM heuristic and Codetta results with 34 columns agree with the ground truth for all but two of these genomes indicating that the translation table data provided by NCBI is erroneous in most of these cases.

**Figure 1:**
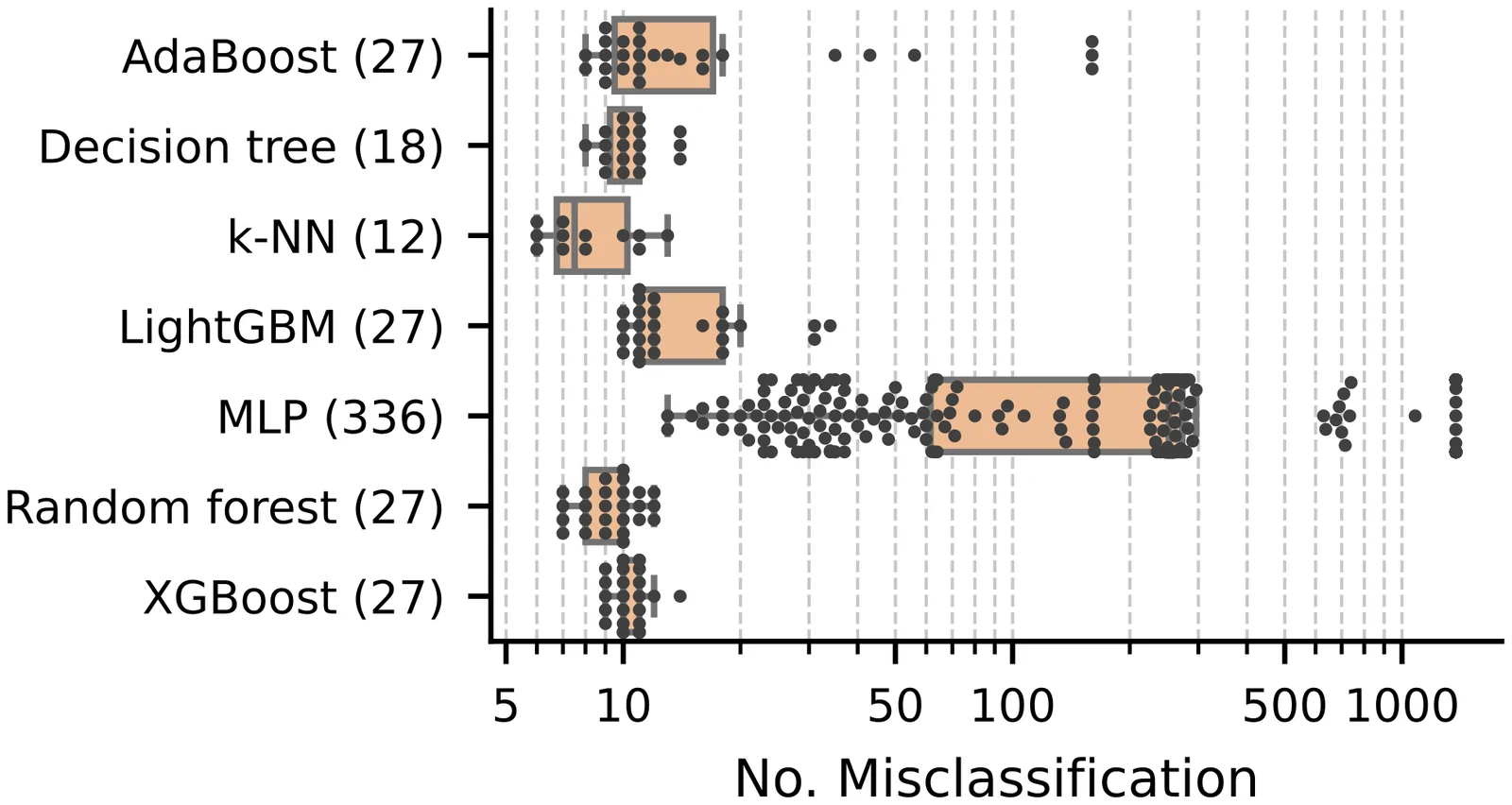
Distribution of misclassifications across combinations of hyperparameter values for seven machine learning classifiers. Results were determined on GTDB R09-RS220 genomes. The total number of parameter combinations tested is indicated in parentheses. The box plots represent the median and interquartile range of performance, while the overlaid swarm plots depict the results for individual combinations.

**Figure 2:**
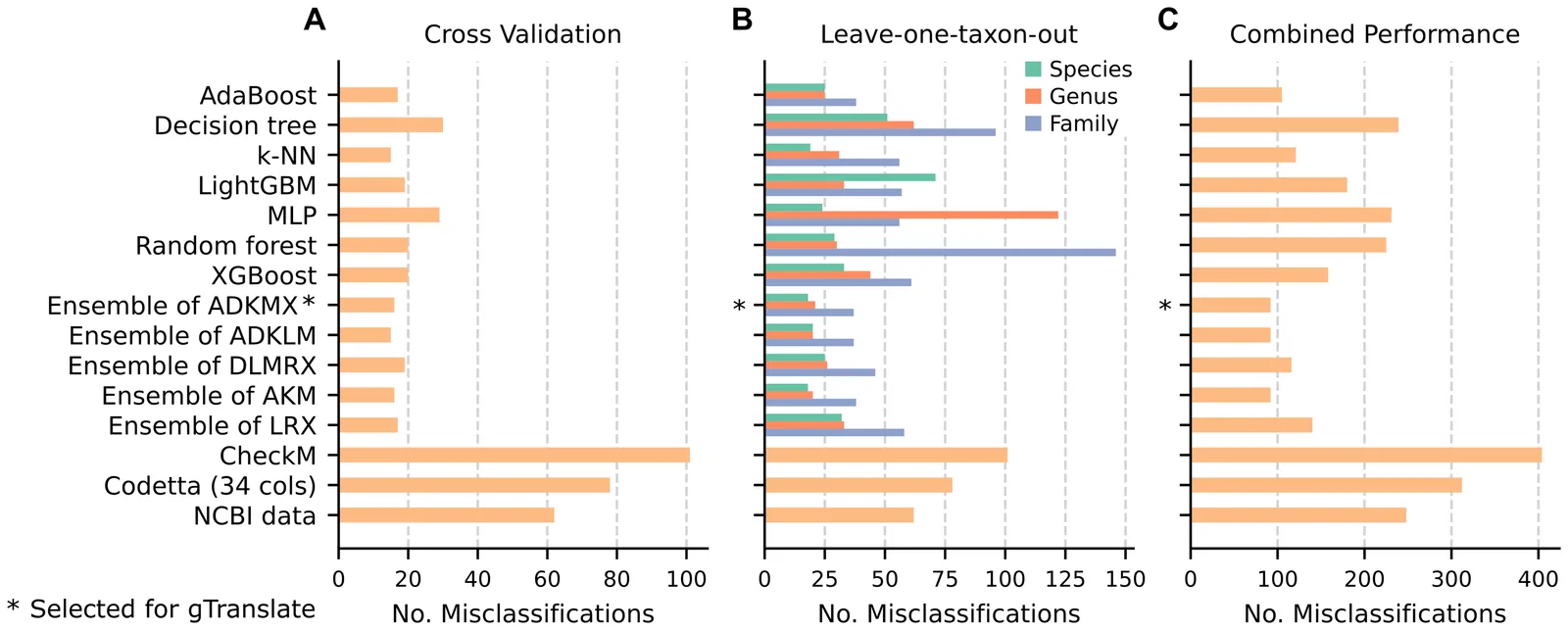
Validation performance on GTDB R09-RS220 genomes. The number of misclassified genomes is provided for both (A) cross validation and (B) leave-one-taxon-out testing methodologies (evaluated at three taxonomic ranks for the ML classifiers), and (C) the combined performance over these two test methodologies. Results are given for individual ML classifiers, the ensemble of classifiers resulting in the best and worst combined performance, the CheckM coding density heuristic, Codetta, and the translation table data provided at NCBI. Codetta results are shown for 34 required columns of support as results requiring only eight columns were substantially worse (444 misclassifications). Note that the single leave-one-taxon-out misclassifications for the non-ML methods were counted three times to calculate the combined performance to be comparable to the ML taxon-specific results. Results for all ensemble classifiers are provided for the cross-validation test in Fig. S2 and leave-one-taxon-out test in Fig. S3. For the ensemble classifiers, A, AdaBoost; D, decision tree; K, *k*-NN; L, LightGBM; M, MLP (multi-layer perceptron); R, random forest; and X, XGBoost.

### Classifier performance on taxonomically novel genomes

The ML classifiers and classifier ensembles were validated using a leave-one-taxon-out procedure to evaluate performance on genomes representing different levels of taxonomic novelty. This was performed by removing a single GTDB taxon from the GTDB R09-RS220 validation set, training on the remaining genomes, and then evaluating the predictions on genomes from the removed taxon. Performance of individual classifiers and ensembles generally decreases with taxonomic novelty (Fig. 2B; Fig. S2), and there was insufficient training data to evaluate classifier performance above the rank of family. For example, leaving out the 1,467 genomes from the order *Mycoplasmatales* leaves only 52 genomes that use translation table 4. Performance of individual classifiers varied substantially; for example, at the rank of species *k*-NN resulted in only 19 misclassifications compared to LightGBM at 71 misclassifications (Table S5: https://doi.org/10.5281/zenodo.20761714). Notably, ensembles of classifiers produced more consistent results. At the species level, the best five ensemble classifier had 18 misclassifications, while the worst had 26. Since the CheckM heuristic, Codetta, and translation table data at NCBI are not dependent on a training set, their predictions are consistent across this validation procedure and produce the same misclassifications as reported in *Performance using Cross Validation*. Notably, all ensemble classifiers outperform these methods.

### Selecting a classifier ensemble

The combined results from the cross validation (Fig. 2A) and leave-one-taxon-out (Fig. 2B) were used to select the ML classifier(s) for gTranslate. Ensembles of 3 or 5 classifiers consistently outperformed individual classifiers (Fig. 2C; Figs. S2 and S3). The best performing individual classifiers were AdaBoost with 105 combined misclassifications and *k*-NN with 121 misclassifications (Table S5: https://doi.org/10.5281/zenodo.20761714). By contrast, there were three classifier ensembles that produced only 92 misclassifications: (i) AdaBoost, *k*-NN, and MLP and (ii) AdaBoost, Decision tree, *k*-NN, MLP, with either XGBoost or LightGBM. We selected the ensemble of five classifiers with XGBoost instead of the equally performing three-classifier ensemble as it provides gTranslate users with additional information since results are provided for each ML classifier. Preference was given to XGBoost over LightGBM as this five-classifier ensemble produced slightly superior performance on the leave-one-taxon-out results at the rank of species, a situation that we expect to occur frequently in real data.

### Performance of gTranslate on GTDB genomes

The expected (test) performance of gTranslate was evaluated by training on the dereplicated set of 356,020 GTDB R09-RS220 genomes and assessing classification accuracy on 116,508 genomes introduced in GTDB R10-RS226. These test genomes represent all new genomes between two annual releases of the GTDB, many of which are from new taxa (Table 1; Table S6: https://doi.org/10.5281/zenodo.20761714). This test set is expected to be indicative of the taxonomic novelty of genomes that gTranslate will be applied to, and hence real-world performance, as gTranslate will be retrained annually with each GTDB release.

**Table 1:** Taxonomic novelty of the 116,508 GTDB R10-RS226 test genomes.

	No. New Taxa		
Rank	Table 11	Table 4	Table 25
Phylum	6	0	0
Class	9	0	0
Order	97	0	0
Family	243	1	0
Genus	3,937	2	8
Species	22,185	64	44

gTranslate correctly predicted the translation table of 116,502 (99.995%) of the 116,508 test genomes with only 6 differing from the expected ground truth (Fig. 3A; Table S2: https://doi.org/10.5281/zenodo.20761714). Of these, five are *Ca.* Stammera capleta genomes that are expected to use table 4 but predicted to use table 11 by gTranslate. Prediction of table 11 is strongly supported by all classifiers comprising the gTranslate ensemble as well as the CheckM heuristic and Codetta. Notably, Codetta strongly supports a UGA stop to tryptophan reassignment for all *Ca.* S. capleta genomes except for these five. Furthermore, all five genomes retain *prfB* (peptide chain release factor RF2), which is typically lost in genomes using genetic code 4 (Bezerra, Guimarães & Santos, 2015), including in the majority of *Ca.* S. capleta genomes (Fig. 4). Unlike most species using genetic code 4, *Ca.* S. capleta genomes do not have a tRNA^Trp^(UCA) codon for recognizing UGA as tryptophan (Manzano-Marín, Kvist & Oceguera-Figueroa, 2023), which precludes using this gene as further genomic evidence in support of *Ca.* S. capleta genomes using genetic code 4 or 11. Nonetheless, most genomic evidence supports the five genomes using genetic code 11 as predicted by gTranslate. Moreover, these genomes form a monophyletic clade basal to all other *Ca.* S. capleta (Fig. 4), a placement observed in previous studies of this species (García-Lozano et al., 2024). This suggests that codon reassignment may have occurred after the basal clade diverged, noting however that genome GCA_036689315.1, which uses genetic code 4, is superficially basal to this group (*i.e.,* without support; Fig. 4).

**Figure 3:**
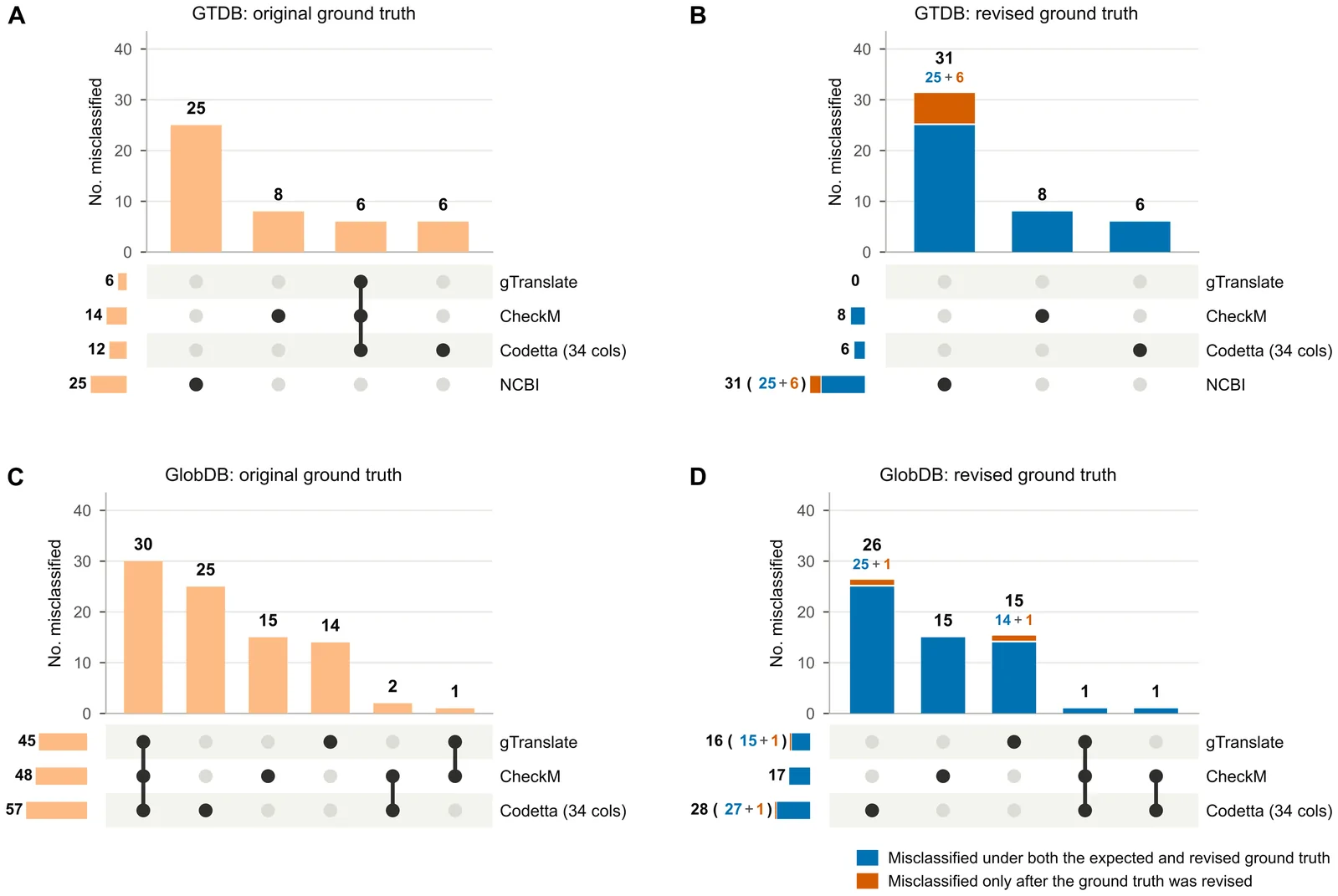
Overlap in misclassifications between gTranslate, the CheckM coding density heuristic, Codetta requiring 34 columns of support, and NCBI translation table data. Misclassifications were determined on the (A and B) 116,508 GTDB R10-RS226 and (C and D) 134,075 GlobDB release 226 test genomes. Results are provided for the (A and C) original ground truth and (B and D) after considering additional evidence suggesting errors in the original ground truth. Bars give the number of genomes misclassified by the combination of methods indicated by the filled dots.

**Figure 4:**
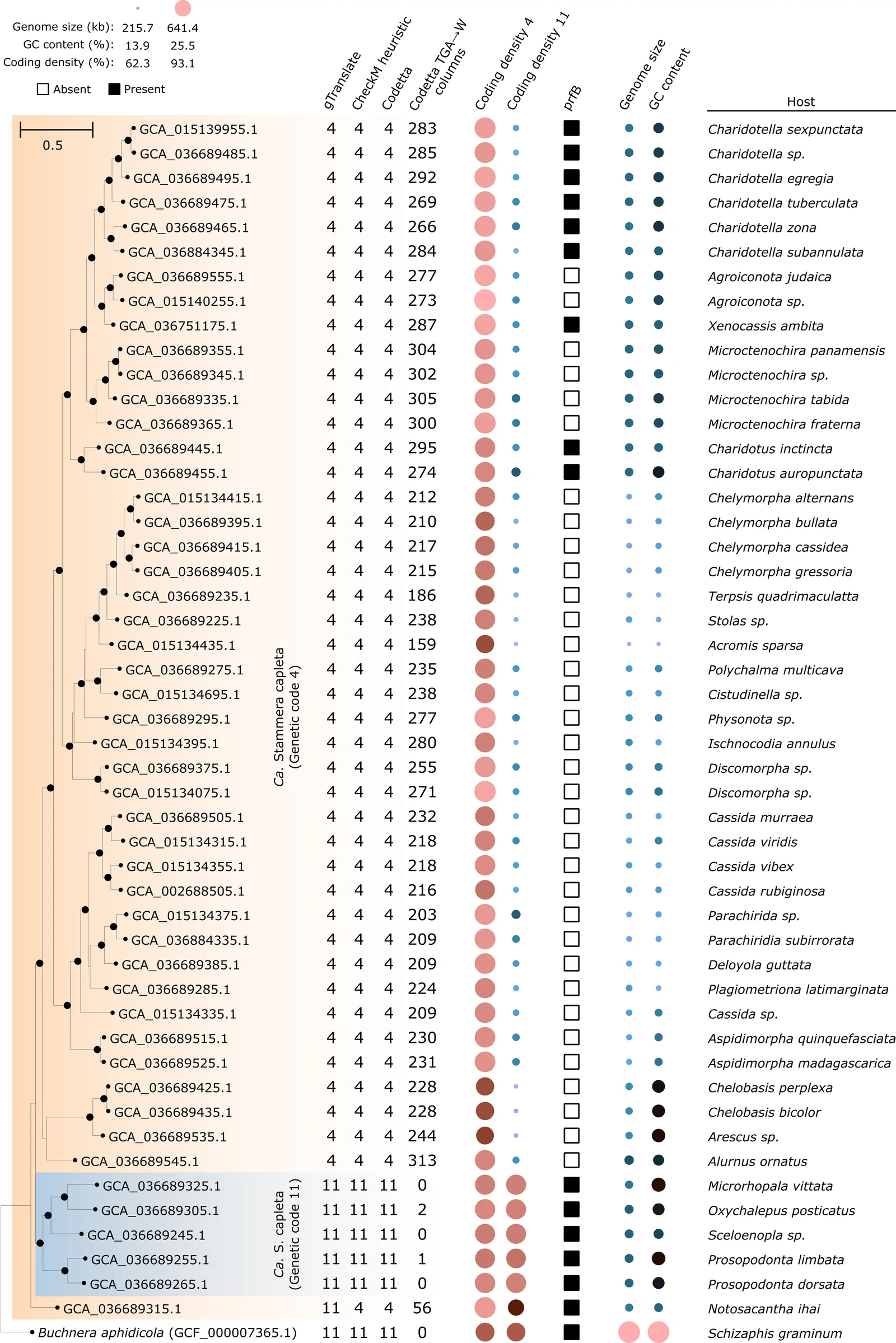
Maximum-likelihood tree for *Ca.* Stammera capleta. The tree was inferred from an alignment of core *Ca.* Stammera capleta genes with IQ-Tree and the GTR+F+R5 evolutionary model. The five S. *capleta* genomes with strong evidence of using genetic code 11 are shown in blue shading. Black dots indicate well supported internal nodes—UFBoot ≥ 95% and SH-aLRT ≥ 80%. *prfB*, peptide chain release factor RF2.

The other discrepant gTranslate prediction is for the *Patescibacteriota* genome GCA_034717395.1, which belongs to the GTDB species JAYELJ01 sp034717395 (Fig. 3A; Table S2: https://doi.org/10.5281/zenodo.20761714). This species is expected to use table 11 but predicted to use table 25. Again, this prediction is strongly supported with four of the five gTranslate classifiers making this prediction, the CheckM heuristic also supporting a reassignment of the UGA stop codon, and Codetta identifying 152 columns with UGA aligned to a tryptophan. GCA_034717395.1 is the only genome in the GTDB order JAYELJ01, and its position on a long branch with minimal support suggests the possibility that the genome is incorrectly placed within the GTDB reference tree and should be assigned to a *Patescibacteriota* order expected to use translation table 25 (Fig. S4).

These results suggest that gTranslate may be making correct predictions for all 116,508 test genomes (Fig. 3B). The CheckM heuristic is in agreement with gTranslate for the six contested cases above, however it makes an additional eight incorrect predictions. All 8 of these misclassifications appear to be legitimate errors and, unlike gTranslate, CheckM is unable to distinguish between genomes using table 4 or 25 (Table S2: https://doi.org/10.5281/zenodo.20761714). Of these misclassifications, seven are incorrectly predicting a UGA stop codon reassignment. The other misclassification is predicting the use of table 11 for a *Mycoplasmatales* genome. Codetta makes 111 or six additional misclassifications when requiring eight or 34 columns of support for a UGA reassignment, respectively. All misclassifications are erroneous UGA reassignments. The six misclassified genomes with ≥34 columns of support are distinct from the misclassifications made by the other methods (Fig. 3B), are predicted in six separate phyla, and include genomes from species that are unlikely to have an unrecognized UGA stop codon reassignment such as *Salmonella enterica* and *Lactococcus cremoris*.

NCBI provides translation table information for 83,262 (71.5%) of the 116,508 test genomes (Table S2: https://doi.org/10.5281/zenodo.20761714). This information differs from the ground truth for 25 genomes (Fig. 3A), 24 of which are assigned to the phylum *Candidatus* Gracilibacteria by NCBI (class *Gracilibacteria* in GTDB R09-RS220). The remaining genome (GCA_035437565.1) is only classified as Bacteria at NCBI, and as such is predicted to use translation table 11. However, this genome belongs to the class JAEDAM01 in GTDB R09-RS220, a taxon comprised of genomes using translation table 25. Notably, gTranslate, the CheckM heuristic, and Codetta agree with the ground truth for all 25 of these genomes (Fig. 3A).

### Performance of gTranslate on GlobDB genomes

Test performance of gTranslate was further evaluated by assessing classification accuracy on 134,075 genomes from GlobDB release 226. The GlobDB test genomes are derived from metagenomic studies not submitted to INSDC, thus representing additional taxonomic novelty relative to GTDB R10-RS226 (Table 2; Table S7: https://doi.org/10.5281/zenodo.20761714). GlobDB is updated with each GTDB release and represents a common use case for gTranslate where large number of genomes of varying taxonomic novelty must be processed.

**Table 2:** Taxonomic novelty of the 134,075 GlobDB test genomes.

	No. New Taxa		
Rank	Table 11	Table 4	Table 25
Phylum	24	0	0
Class	119	0	0
Order	530	0	0
Family	6,643	10	13
Genus	25,776	26	99
Species	90,619	32	228

gTranslate correctly predicts the translation table of 134,030 (99.97%) of the GlobDB test genomes (Fig. 3C; Table S3: https://doi.org/10.5281/zenodo.20761714). However, like the results on the GTDB R10-RS226 test genomes, the majority of the 45 putative misclassifications appear to be errors in the ground truth due to previously unrecognized stop code reassignments or taxonomic misclassifications (Fig. 3D). The most notable of these are 12 genomes from the JAKLIH01 family and 10 genomes from the GCA-2747955 genus in the class *Minisyncoccia* where genomic evidence indicates a reassignment of the UGA stop codon to tryptophan (Fig. 5; Table S8: https://doi.org/10.5281/zenodo.20761714). These 22 genomes have large numbers of columns where UGA aligns with a tryptophan in conserved proteins, mostly have an identified tRNA^Trp^(UCA) gene (*i.e.,* the anticodon of UGA), and lack a *prfB* gene consistent with organisms using a reassigned UGA stop codon. These are the first organisms in the *Patescibacteriota* with a predicted UGA-to-tryptophan reassignment making this the first bacterial phylum to use three genetic codes.

**Figure 5:**
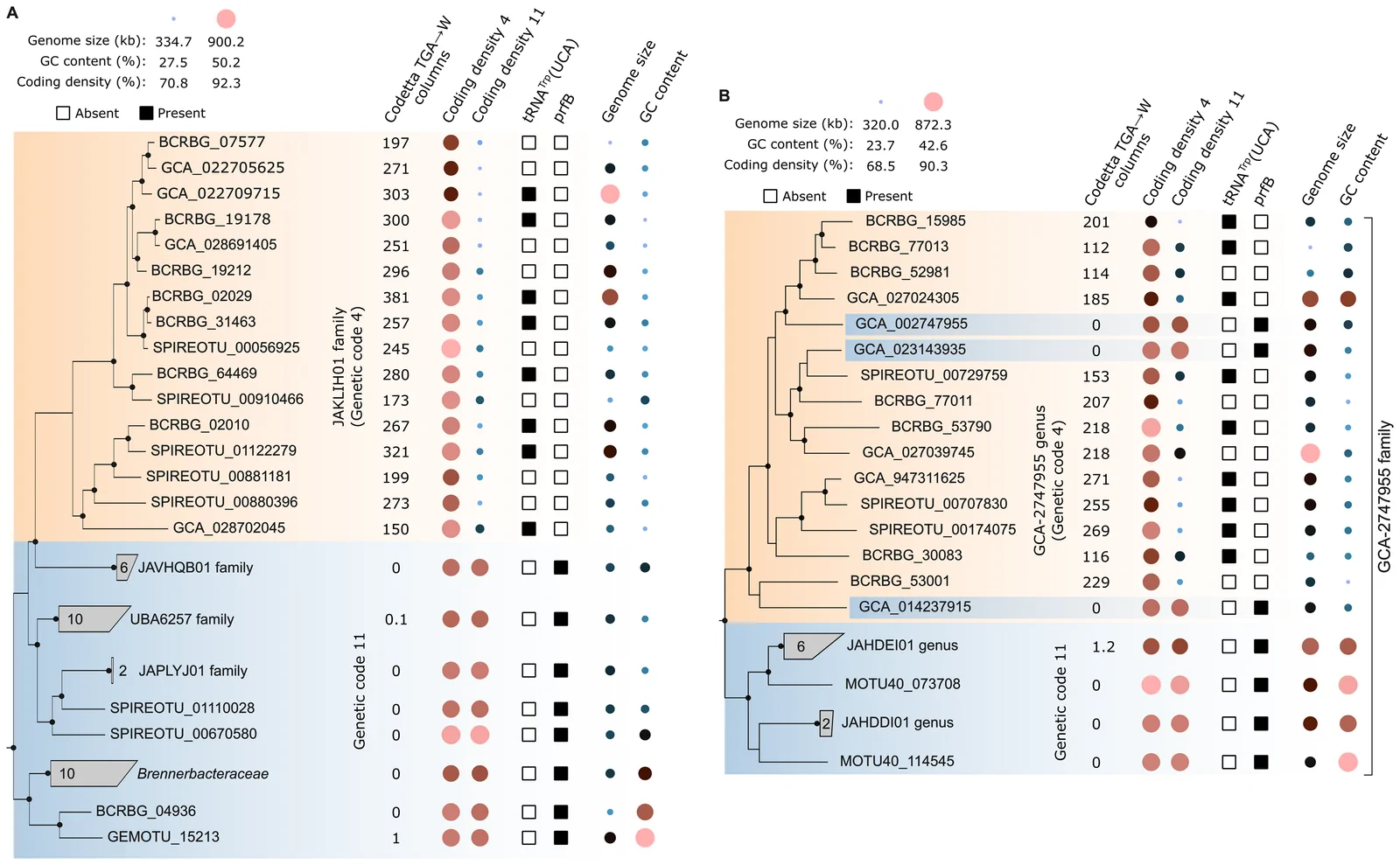
Genomic evidence supporting a UGA stop-to-tryptophan reassignment in *Patescibacteriota*. This stop reassignment was identified in two class *Minisyncoccia* lineages, specifically the (A) family JAKLIH01 and (B) genus GCA-2747955. The maximum-likelihood trees were inferred from a set of 120 bacterial marker genes using IQ-Tree with the LG+F+I+R10 evolutionary model. Data is provided for the 16 JAKLIH01 and 14 GCA-2747955 genomes in GlobDB release 226 along with genomes from closely related families (A) or genera (B). The three genomes in the GCA-2747955 genus shown with blue highlighting still use genetic code 11 despite being in a lineage otherwise comprised of genomes with the UGA stop codon reassigned to tryptophan. Genomes with identifiers that do not start with GCA are part of the GlobDB test set, whereas genomes starting with GCA are part of GTDB. Taxa were dereplicated to at most 10 genomes, with taxa represented by a single genome labelled using the GlobDB genome identifier. Genomic properties for taxa with multiple genomes indicate the average across these genomes. The *prfB* gene (peptide chain release factor RF2) was identified in all genomes using genetic code 11 except for a single *Brennerbacteraceae* genome. Black dots on internal branches indicate strong support with UFBoot ≥ 95% and SH-aLRT ≥ 80%.

There are also two genomes belonging to *Eggerthellaceae* with a ground truth of table 11 predicted to use table 4 by gTranslate, the CheckM heuristic, and Codetta (Table S3: https://doi.org/10.5281/zenodo.20761714), consistent with our recent report of UGA-to-tryptophan reassignment in members of this bacterial family (Parks et al., 2026). Similar to the GCA_034717395.1 genome in the GTDB R10-RS226 test set, there are five genomes assigned to the *Patescibacteriota* order GCA-2401425 with a ground truth of 11, but genomic evidence that they use table 25 (Table S3: https://doi.org/10.5281/zenodo.20761714) and thus are likely taxonomically misclassified (Fig. S4). Finally, there is a single *Burkholderiaceae* genome that was assigned to a novel genus by GlobDB and thus has a default ground truth of table 11. However, this genome is assigned to the genus *Zinderia* when using GTDB-Tk with the GTDB R10-RS226 reference data, contains a tRNA^Trp^(UCA) gene, lacks a *prfB* gene, is classified to table 4 by the CheckM heuristic and has 30 columns identified by Codetta in support of a UGA-to-tryptophan reassignment (Table S3: https://doi.org/10.5281/zenodo.20761714). This suggests that this genome uses translation table 4.

gTranslate predictions appear to be legitimately incorrect for 13 genomes in the *Patescibacteriota* class JAEDAM01 (Fig. 3D; Table S3: https://doi.org/10.5281/zenodo.20761714). These genomes are all expected to use table 25 based on their taxonomic classification, and the Codetta results support this assignment. However, gTranslate predicts table 4 for 11 of these genomes and table 11 for the remaining two genomes. In addition, there are two *Mycoplasmatales* genomes that gTranslate incorrectly predicts as using table 25 or 11 instead of table 4 (Table S3: https://doi.org/10.5281/zenodo.20761714). Surprisingly, the CheckM heuristic and Codetta both predict table 11 along with gTranslate for the BCRBG_74280 *Mycoplasmatales* genome; however, this genome has a tRNA^Trp^(UCA) gene and lacks a *prfB* gene suggesting the ground truth is correct. There are also two *Minisyncoccia* genomes that are predicted to have a reassigned stop codon according to the CheckM heuristic and Codetta (Table S3: https://doi.org/10.5281/zenodo.20761714). Genomic evidence supports a stop reassignment for one of these genomes and indicates it uses table 4 as identified for other *Minisyncoccia* lineages in this study. The other reassignment is to table 25 but the absence of a tRNA-Gly(UCA) and presence of the *prfB* gene do not support this recoding, making it more likely that this misclassification is due to the relatively poor quality of the genome assembly (74.4% complete; 4.2% contamination).

The 15 and 26 misclassifications made only by the CheckM heuristic and Codetta, respectively, appear to be legitimate errors (Fig. 3C; Table S3: https://doi.org/10.5281/zenodo.20761714). All 15 of the misclassifications exclusive to the CheckM heuristic are for genomes with a predicted UGA stop codon reassignment. Notably, these genomes have abnormally low coding density when genes are called using table 4/25 (mean = 81.3%) or table 11 (75.6%), that may be in part due to a high number of contigs in the assemblies (mean = 506). Most of these genomes have a *prfB* gene (11 of 15) and all lack a tRNA-Trp(UCA) or tRNA-Gly(UCA) gene indicating that the ground truth is correct. Of the 26 misclassifications exclusive to Codetta, 25 have a ground truth of table 11, with 23 and two genomes predicted to use table 25 and 4, respectively. Most of these genomes (21 of 25) have <100 columns supporting a stop codon reassignment, mostly have a *prfB* gene (22 of 25), and all genomes lack a tRNA-Trp(UCA) or tRNA-Gly(UCA) gene indicating that the ground truth of table 11 is likely correct.

## Discussion

gTranslate provides robust predictions of translation tables used by bacterial genomes, making only 16 misclassifications (99.994% accuracy; Figs. 4B and 4D) across the 250,583 test genomes in GTDB R10-RS226 (Parks et al., 2026) and GlobDB release 226 (Speth et al., 2024). Most misclassifications (12 of 16) were a result of incorrectly distinguishing between table 4 and 25 that both reassign the UGA stop codon, resulting in an accuracy of 98.9% across the 1,077 genomes that do not use the standard bacterial translation table 11 (Tables S2 and S3). Notably, gTranslate has better accuracy than the taxonomically-defined ground truth as five *Ca.* Stammera capleta (Fig. 4) and 22 *Minisyncoccia* genomes (Fig. 5) have genomic evidence indicating the use of a translation table that conflicted with taxonomic expectations. In addition, we attributed nine putative gTranslate misclassifications to inaccurate taxonomic classifications either due to lack of taxonomic resolution provided by automated methods (Table S3: https://doi.org/10.5281/zenodo.20761714) or misplacement in reference phylogenies (Fig. S4). The challenge in using taxonomic classifications to establish the translation table for genomes is further illustrated by information provided at NCBI being incorrect for 31 of the 83,262 (99.96% accuracy; Fig. 3B) GTDB R10-RS226 genomes where NCBI translation table information was available.

gTranslate outperforms the CheckM coding density heuristic and Codetta that had 25 and 34 misclassifications across the 250,583 test genomes, respectively (Figs. 4B and 4D). Furthermore, gTranslate can distinguish between table 4 and 25, whereas the CheckM heuristic can only indicate that a UGA stop codon reassignment has occurred. This is particularly notable as only 4 of the 16 misclassifications made by gTranslate are due to incorrectly distinguishing table 11 from a UGA stop codon reassignment. While Codetta is treated as a classifier for predicting translation table 4, 11, and 25 in this manuscript (see *Methods*), it is a more general tool that aims to identify the most likely amino acid encoded by each codon (Shulgina & Eddy, 2021). The Codetta results reported here only consider reassignment of the UGA stop codon and do not consider any additional insights (or misclassifications) that may arise by considering the reassignment of other codons. gTranslate is a focused tool that allows for more computationally efficient prediction of established translation tables with higher accuracy than Codetta. For reference, gTranslate is ∼100× times more computationally efficient than Codetta requiring only ∼20 s per genome per CPU on an Intel Xeon E5-2640 v4 2.40 GHz (released in 2016). In practical terms, Codetta is challenging to apply to large datasets as illustrated by it requiring five weeks of computation when using 96 E5-2640 CPUs to process the 116,508 new genomes in GTDB R10-RS226.

The results on the GTDB and GlobDB test genomes indicate gTranslate can accurately predict the translation table of genomes with varying levels of taxonomic novelty (Tables 1 and 2). Nonetheless, the performance of gTranslate is best when it is trained on genomes similar to those for which it is making predictions as illustrated by the leave-one-taxon-out validation analysis (Fig. 2B; Fig. S2). A benefit of basing gTranslate on GTDB genomes is that this resource will be updated annually allowing for regular retraining of gTranslate with each GTDB release. Annual updating will also allow incorporation of new stop codon reassignments such as those reported in this and other recent studies (Nakayama et al., 2025; Parks et al., 2026).

Direct genomic evidence in support of a UGA stop codon reassignment such as the presence of a tRNA^Trp^(UCA) (McCutcheon, McDonald & Moran, 2009) or tRNA^Gly^(UCA) (Campbell et al., 2013), or lack of *prfB* (Bezerra, Guimarães & Santos, 2015) is considered throughout this study. While these are key molecular signatures of a reassigned UGA stop codon, they are not well suited for predicting the translation table used by individual genomes. This is exemplified by strains of *Ca.* Stammera capleta (Salem et al., 2017) that use genetic code 4 despite always lacking a tRNA^Trp^(UCA) and containing a *prfB* in some assemblies (Fig. 4). More generally, translation table prediction must be robust to partial genome assemblies as these predictions are an initial step in bioinformatic programs such as CheckM (Chklovski et al., 2023) and GTDB-Tk (Chaumeil et al., 2022), which are applied to large numbers of genomes, including large-scale studies consisting of incomplete metagenome-assembled genomes. Molecular signatures are therefore best interpreted in a phylogenomic context, where evidence can be evaluated across multiple (potentially incomplete) genomes alongside additional genomic indicators, such as UGA codon alignment in conserved proteins and coding density under different translation tables.

gTranslate is currently limited to prokaryotic genomes and predicting translation table 11, 4, or 25 where UGA is a stop codon or translated as tryptophan or glycine, respectively. However, other reassignments have been computationally inferred and experimentally verified in some cases. Application of Codetta to over 250,000 prokaryotic genomes revealed a number of putative arginine sense codon reassignments (Shulgina & Eddy, 2021), though these have not yet been experimentally verified (Zürcher et al., 2022). More recently, it has been computationally predicted that a phylogenetically sporadic set of archaeal genomes likely have genome-wide reassignment of the UAG stop codon to pyrrolysine, which has been confirmed by proteomic analysis for two genomes (Kivenson et al., 2025). Notably, this UAG-to-pyrrolysine reassignment is more difficult to detect as it does not result in reduced coding density when genes are identified using translation table 11 as observed for UGA stop codon reassignments. Methods have also been proposed for handling specific fungal (Mühlhausen & Kollmar, 2014) or mitochondrial reassignments (Abascal, Zardoya & Posada, 2006; Noutahi et al., 2017). These all represent promising directions for future development of gTranslate that would build upon the approach developed in this study. For example, we anticipate incorporating identification of genome-wide UAG stop codon to pyrrolysine reassignments in the next release of gTranslate.

## Conclusion

gTranslate provides a machine learning-based approach for the accurate prediction of prokaryotic translation tables. It improves upon the coding density-based heuristic used by existing bioinformatics software by being able to distinguish between translation table 4 and 25. By utilizing an ensemble of five classifiers, gTranslate achieves >99.99% accuracy across diverse test datasets while having approximately 100 times greater computational efficiency than Codetta. Use of gTranslate led to the discovery of a basal *Ca.* Stammera capleta lineage that uses the standard bacterial genetic code and the first instances of UGA-to-tryptophan reassignment in the *Patescibacteriota* class *Minisyncoccia*. These findings identify *Patescibacteriota* as the first bacterial phylum to use table 4, 11, and 25. Although gTranslate is currently limited to the most prevalent prokaryotic codes, we anticipate that annual retraining with GTDB updates will facilitate the future incorporation of novel variations, such as the recent genome wide UAG-to-pyrrolysine reassignment identified in Archaea. Ultimately, gTranslate offers a scalable and robust solution for the high-throughput prediction of translation tables for prokaryotic genomes, ensuring that genetic code variation is accurately captured in large-scale studies and genomic repositories.

##  Supplemental Information

10.7717/peerj.21825/supp-1 Figure S1 Pairwise distributions of eight genomic features across translation tables Pairwise scatter plot of 8 genomic features computed for 356,018 prokaryotic genomes from GTDB R09-RS220. Off-diagonal panels display scatter plots for each pair of features, with points coloured by their assigned translation table: table 11 (light blue), table 25 (red), and table 4 (dark blue). Diagonal panels display kernel density estimates (KDEs) for each feature, stratified by translation table and independently normalised to a peak density of one to enable visual comparison across groups despite substantial differences in group size. Feature abbreviations: CD4, coding density under translation table 4 (equivalently 25); CD11, coding density under translation table 11; CD4–CD11, difference in coding density between tables 4 and 11; Trp, tryptophan; Gly, glycine.

10.7717/peerj.21825/supp-2 Figure S2 Cross-validation performance of classifiers and ensembles Cross validation performance of the seven ML classifiers, ensembles of 3 (A) and 5 (B) classifiers, CheckM heuristic, Codetta with 8 and 34 columns of support, and NCBI. Results indicate the number of misclassifications across all 5 folds made on genomes with a ground truth of table 11 (grey), table 4 (red), or table 25 (blue). Recoding misclassifications (purple; B) indicate cases where the ground truth is table 4 or 25 but the classifier predicts table 11.

10.7717/peerj.21825/supp-3 Figure S3 Leave-one-taxon-out performance of classifiers and ensembles Leave-one-taxon-out performance at the rank of family, genus, and species across seven ML classifiers, ensembles of five classifiers, CheckM heuristic, Codetta with 34 columns of support, and NCBI. Results indicate the number of misclassifications across on genomes with a ground truth of table 11 (grey), table 4 (red), or table 25 (blue).

10.7717/peerj.21825/supp-4 Figure S4 Phylogenetic context of Gracilibacteria lineages JAYELJ01 sp034717395 and GCA-2401425 GTDB R10-RS226 reference bacterial tree illustrating the position of the JAYELJ01 sp034717395 genome GCA_034717395.1 and genomes in the order GCA-2401425 (shown in red). The ground truth for these genomes is translation table 11 because of their position within the class Gracilibacteria. gTranslate, CheckM, and Codetta all predict these genomes use translation table 25 as would be expected for a genome in the class JAEDAM01, which is sister to the Gracilibacteria. In addition, all these genomes lack the translational release factor RF2 gene. The relatively low non-parametric bootstrap support values between these genomes and JAEDAM01 suggest they may be incorrectly placed within the GTDB reference tree and could belong to class JAEDAM01.

## Additional Information and Declarations

### Data Availability

The following information was supplied regarding data availability: The code is available at GitHub and Zenodo: - https://github.com/cmc-aau/gTranslate. - Chaumeil, P.-A., Hugenholtz, P., & Parks, D. (2026). gTranslate manuscript supplementary files (Version v1.2.0) [Dataset]. Zenodo. https://doi.org/10.5281/zenodo.20761714.
